# The mechanism of liver X receptor regulates the balance of glycoFAsynthesis and cholesterol synthesis in clear cell renal cell carcinoma

**DOI:** 10.1002/ctm2.1248

**Published:** 2023-05-03

**Authors:** Qifei Wang, Wei Zhang, Xiaochen Qi, Jiayi Li, Yuanxin Liu, Quanlin Li, Yingkun Xu, Guangzhen Wu

**Affiliations:** ^1^ Department of Urology The First Affiliated Hospital of Dalian Medical University Dalian China; ^2^ School of Life Sciences and Biotechnology Shanghai Jiao Tong University Shanghai China; ^3^ State Key Laboratory of Medical Genomics and Shanghai Institute of Hematology Ruijin Hospital, Shanghai Jiao Tong University School of Medicine Shanghai China; ^4^ School of Business Hanyang University Seoul Republic of Korea; ^5^ Department of Breast and Thyroid Surgery The First Affiliated Hospital of Chongqing Medical University Chongqing China; ^6^ Department of Urology Shandong Provincial Hospital Cheeloo College of Medicine Shandong University Jinan China

Dear Editor,

Renal cell carcinoma (RCC) is a prevalent cancer, ranking among the top ten worldwide cancers and accounting for 2% of all cancers globally, with an increasing trend annually.^1^ Clear cell renal cell carcinoma (ccRCC), arising from proximal renal tubular cells, is highly malignant, accounting for all genitourinary tumours.^2^ Approximately 30% of ccRCC cases tend to metastasize, with poor prognosis.^3^ Early diagnosis of ccRCC is challenging as clinical manifestations are often elusive, and it lacks effective tumour markers while being insensitive to radiotherapy and chemotherapy. Therefore, identifying key molecules for diagnosis and treatment is a critical research focus.^4^ Research has established a link between cellular metabolic disorders and tumour occurrence and progression, with abnormal glucose and lipid metabolism, including cholesterol metabolism, emerging as key factors in ccRCC.^5^, ^6^, ^7^, ^8^ This study employed laboratory experiments and extensive biological information analysis to elucidate the potential mechanism of glycoFAsynthesis and cholesterol biosynthesis pathways in ccRCC.

In the development and progression of ccRCC, small changes in tumour cells lead to the accumulation of metabolites, producing atypical metabolites.^9^ Metabolomics can study metabolic pathways in ccRCC, aiding in ID'ing novel therapeutic targets.^10^ We found that glucose, glucose‐6‐phosphate, fructose‐6‐phosphate, lactate, stearate, oleate, palmitate, palmitoleate and cholesterol levels were higher in ccRCC than kidney tissues (Figure 1A–I). Lactate levels were higher in high‐stage and high‐grade ccRCC tumours than in low‐stage and low‐grade tumours (Figure 1J,K). We used gene expression profiles for glycoFAsynthesis and cholesterol synthesis pathways to classify ccRCC patients into three clusters (C1, C2, and C3) (Figure 1L), and generated a four‐quadrant diagram using Z‐scores of these pathways' gene expression profiles (Figure 1M). Relevant data are in Table S1. We stratified ccRCC patients into quiescent, glycoFAsynthesis, cholesterol, and mixed subgroups. We explored genetic differences, drug sensitivity, and clinical outcomes in these subgroups. The heat map showed upregulated glycoFAsynthesis genes in glycoFAsynthesis and mixed subgroups, while cholesterol synthesis genes were highly expressed in cholesterol and mixed subgroups (Figure 1N and Table S2). Based on pathological features and survival information of ccRCC patients, we developed a corresponding survival curve. The glycoFAsynthesis subgroup had the worst prognosis among almost all pathological types (Figure 1O–Q and Figure S1A–F). In contrast, the performance of the cholesterol synthesis subgroup was distinct. For instance, the cholesterol synthesis subgroup in the T1 and T2 groups demonstrated a better prognosis, whereas the cholesterol synthesis subgroup in the T3 and T4 groups exhibited a poor prognosis. Cholesterol synthesis had significantly different prognostic implications for patients in the early and late stages of ccRCC. Drug sensitivity analysis from the GDSC database showed that pazopanib, temsirolimus, lapatinib, and bosutinib were more effective in the glycoFAsynthesis subgroup, while rapamycin, vinblastine, gefitinib, and metformin were more effective in the cholesterol subgroup (Figure 1R–Y). Submap analysis indicated that the cholesterol subgroup may be more responsive to CTLA‐4 inhibitors (*p*‐value = .01) (Figure 1Z).

**FIGURE 1:**
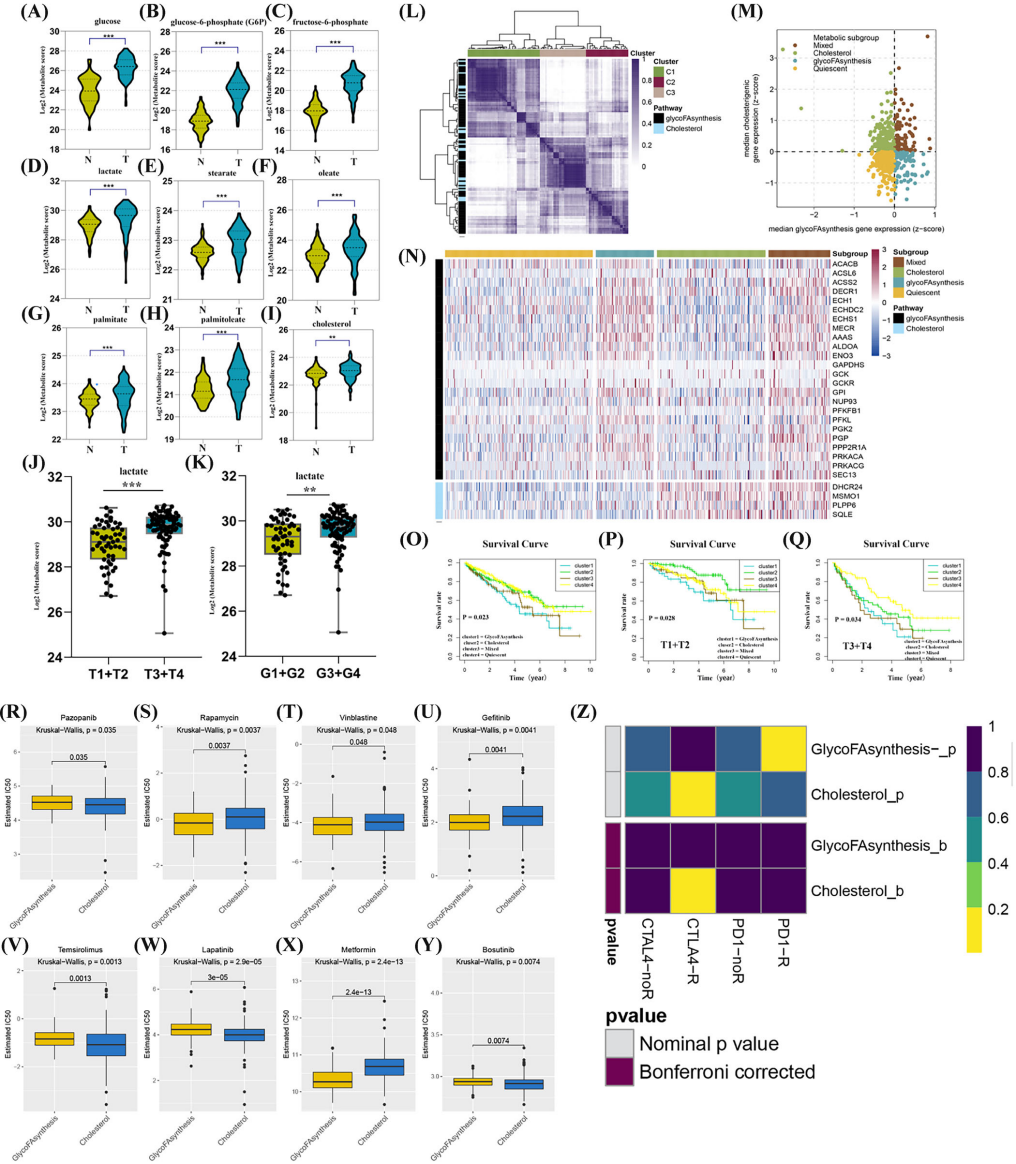
Stratification of clear cell renal cell carcinoma (ccRCC) tumours based on the expression of glycoFAsynthesis pathway and cholesterol biosynthesis pathway genes. (A–I) Violin plot of the difference in the content of main products of glycoFAsynthesis and cholesterol biosynthesis pathways between ccRCC tumour tissue and normal kidney tissue. The main products are glucose, glucose‐6‐phosphate, fructose‐6‐phosphate, lactate, stearate, oleate, palmitate, palmitoleate, and cholesterol. (J) This box plot shows the lactate level in T1+T2 and T3+T4 ccRCC tumours. (K) This box plot shows the lactate level in G1+G2 and G3+G4 ccRCC tumours. **P < 0.01, ***p < .001. (L) Heat map depicting consensus clustering solution for glycoFAsynthesis pathway and cholesterol biosynthesis pathway genes in ccRCC samples. (M) Scatter plot showing median expression levels of co‐expressed glycoFAsynthesis pathway (x‐axis) and cholesterol biosynthesis pathway (y‐axis) genes in each ccRCC sample. Metabolic subgroups were assigned based on the relative expression levels of glycoFAsynthesis pathway and cholesterol biosynthesis pathway genes. (N) The heat map depicts the expression levels of co‐expressed glycoFAsynthesis pathway and cholesterol biosynthesis pathway genes across each subgroup. (O–Q) Kaplan–Meier survival analysis of patients with All, T1+T2 and T3+T4 ccRCC stratified by metabolic subgroup. P values are as shown. This shows the differences in drug sensitivity, differences in immune correlation, and differences in the expression of cancer/tumour suppressor genes between the two ccRCC subgroups of glycoFAsynthesis and cholesterol biosynthesis. (R–Y) Box plots show the IC50 values of pazopanib, rapamycin, vinblastine, geftinib, temsirolimus, lapatinib, metformin, and bosutinib, respectively, in the two subgroups of ccRCC patients with glycoFAsynthesis and cholesterol biosynthesis. (Z) Submap analysis shows the correlation between inhibitors corresponding to the immune checkpoints (PD1‐R, PD1‐noR, CTLA4‐R and CTAL4‐noR) and the two ccRCC subgroups of glycoFAsynthesis and cholesterol biosynthesis. From the perspective of bioinformatics and metabolomics, the results suggest that glycoFAsynthesis and cholesterol biosynthesis have great changes in ccRCC. These two changes are likely to have an important impact on the occurrence and development of ccRCC.

Liver X receptor (LXR) molecules play a vital role in glucose and lipid metabolism, as well as cholesterol metabolism. To study the relationship between glycoFAsynthesis, cholesterigenic pathways, and LXR, we classified LXR into NR1H2 (LXRβ) and NR1H3 (LXRα) subtypes.^7^ Heat maps showed a strong positive correlation between glycoFAsynthesis and NR1H2 (Figure 2A,B). Multivariate GSEA revealed that NR1H2 was related to osteoblast differentiation and regucalcin activation in proximal tubule epithelial kidney cells (Figure 2C), while NR1H3 was linked to endothelin pathways and regulation of the actin cytoskeleton (Figure 2D). Violin diagrams demonstrated that the enrichment scores of NR1H2 and NR1H3 in the four subgroups according to the cholesterol synthesis pathway were significantly lower in the cholesterol subgroup than in the glycoFAsynthesis subgroup (Figure 2E,F). To investigate LXR's role in ccRCC, ACHN renal cancer cells were treated with LXR's inverse agonist SR9243. Sequencing results showed differential expression of many genes in the SR9243 group compared to the control group (Table S3). The differentially expressed genes were subjected to gene ontology (GO) and Kyoto Encyclopedia of Genes and Genomes (KEGG) analyses. BP analysis results indicated that these differentially expressed genes (DEGs) were associated with RNA catabolic process and translational initiation. Furthermore, DEGs were associated with focal adhesion and cell‐substrate junction, according to CC analysis. In addition, DEGs were associated with cadherin binding and cell adhesion molecule binding according to MF analysis (Figure 2G). KEGG analysis showed enrichment in amyotrophic lateral sclerosis and human papillomavirus infection (Figure 2H). GSVA analysis suggested SR9243 may trigger mTORC1 and TNF‐α signalling via NF‐κB and MYC targets v1 (Figure 2K). Heatmaps and a volcano map were generated to display differentially expressed genes related to cholesterol transport, glycolysis, fatty acid synthesis, and cholesterol synthesis (Figures 2I,J). A potential mechanism was proposed, demonstrating how the LXR inverse agonist SR9243 exerts biological effects in ccRCC by regulating glucose and lipid metabolism using differentially expressed genes (Figure 2L). In vivo experiments were done with LXR agonist LXR623 in nude mice, and the treatment group showed a significant reduction in tumour weight and volume (Figure 2N–Q). Then, LXR623 was used to treat ACHN renal cancer cells, resulting in differentially expressed genes compared to the control (Table S4). GO and KEGG analyses of DEGs revealed that BP analysis showed links to the regulation of cell cycle phase transition and organelle fission. CC analysis revealed links to focal adhesion and cell‐substrate junction, while MF analysis showed links to cadherin binding and DNA‐binding transcription factor binding (Figure 2R). KEGG analysis showed relevance to amyotrophic lateral sclerosis and Huntington's disease (Figure 2S). GSVA analysis results suggested LXR623's role in ccRCC by activating MYC targets v1 and E2F targets (Figure 2V). Additionally, a heatmap displaying the expression of cholesterol transport, glycolysis, fatty acid synthesis, and cholesterol synthesis‐related genes before treatment with LXR623 and control groups was generated (Figure 2T–X). A volcano map displaying the differentially expressed genes was also generated (Figure 2Y). Finally, a potential mechanism of LXR623 was proposed in regulating glucose and lipid metabolism through differentially expressed genes (Figure 2M), suggesting LXR623 promotes cholesterol efflux and glycolipid synthesis.

**FIGURE 2:**
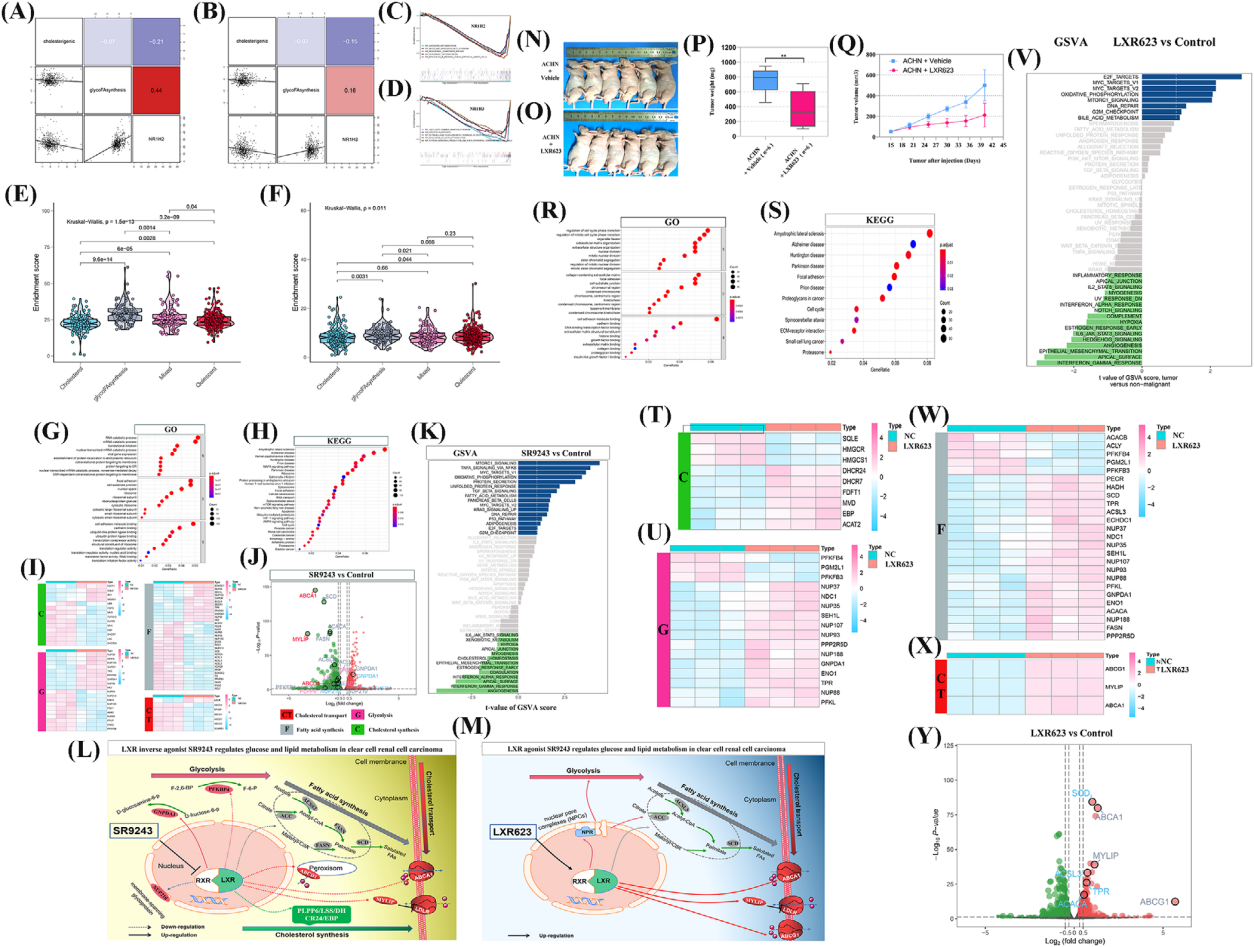
(A) This picture shows the co‐expression between NR1H2 (LXRβ) and the two clear cell renal cell carcinoma (ccRCC) subgroups of glycoFAsynthesis and cholesterol biosynthesis. (B) This shows the co‐expression between NR1H3 (LXRα) and the two ccRCC subgroups of glycoFAsynthesis and cholesterol biosynthesis. (C) This shows the results of multivariate GSEA analysis of NR1H2 (LXRβ) in ccRCC. (D) This shows the results of multivariate GSEA analysis of NR1H3 (LXRα) in ccRCC. (E) Violin plot of the enrichment score of NR1H2 (LXRβ) in the four ccRCC subgroups. (F) Violin plot of the enrichment score of NR1H3 (LXRα) in the four ccRCC subgroups. P values are as shown. (G) The bubble chart shows the gene ontology (GO analysis results of differentially expressed genes between the SR9243 treatment group and the negative control group, including BP, CC and MF. (H) This shows the Kyoto Encyclopedia of Genes and Genomes (KEGG) analysis results of differentially expressed genes between the SR9243 treatment group and the negative control group. (I) Heat map shows the expression of differentially expressed genes enriched in the cholesterol biosynthesis pathway, glycolysis pathway, fatty acid synthesis pathway, and cholesterol transport pathway in ccRCC. Among them, “C” means cholesterol biosynthesis pathway, “G” means glycolysis pathway, “F” means fatty acid synthesis pathway, and “CT” means cholesterol transport pathway. (J) Volcano plot of the differentially expressed genes between the SR9243 treatment group and the negative control group. (K) GSVA shows the difference in biological pathway activity between the tumour cells in the SR9243 treatment group and the normal control group. (L) The schematic diagram shows the process that liver X receptor (LXR) inverse agonist SR9243 regulates glucose and lipid metabolism in clear cell renal cell carcinoma. (M) The schematic diagram shows the process that LXR agonist LXR623 regulates glucose and lipid metabolism in clear cell renal cell carcinoma. (N–Q) The picture shows the appearance of nude mice between the negative control group and the LXR623 treatment group in the in vivo tumour formation experiment, as well as the box plot showing the tumour weight and the line graph of the tumour volume change between the two groups. (R) Bubble chart shows the GO analysis results of differentially expressed genes between the LXR623 treatment group and the negative control group, including BP, CC, and MF. (S) Bubble chart shows the KEGG analysis results of differentially expressed genes between the LXR623 treatment group and the negative control group. (V) GSVA shows the difference in biological pathway activity between the tumour cells in the LXR623 treatment group and the normal control group. (T, U, W, X) Heat map shows the expression of differentially expressed genes enriched in the cholesterol biosynthesis pathway, glycolysis pathway, fatty acid synthesis pathway, and cholesterol transport pathway in ccRCC. Among them, “C” means cholesterol biosynthesis pathway, “G” means glycolysis pathway, “F” means fatty acid synthesis pathway, and “CT” means cholesterol transport pathway. (Y) The volcano plot shows the differentially expressed genes between the LXR623 treatment group and the negative control group.

The study used the mulberry map to show the relationship between gene expression, LXR drugs, and key pathways (glycolysis, fatty acid synthesis, cholesterol transport and cholesterol synthesis) (Figure 3A). Four‐quadrant diagram highlighted genes associated with fatty acid synthesis and cholesterol transport (Figure 3B). The rainfall plot identified NUP210, SCD and ABCG1 as key players in ccRCC lipid metabolism (Figure 3C). The schematic diagram showed LXR drugs inducing cell death by disrupting biomembrane integrity (Figure 3D). Furthermore, a Venn diagram was utilized to identify the genes involved in the lipid synthesis pathway of biomembranes and the DEGs caused by SR9243 and LXR623 treatment. The analysis revealed that A4GALT, GAL3ST1, SPTLC2, ST3GAL2, B4GALT5 and ABCA2 were shared by all three gene sets (Figure 3E). The heat map showed these six genes had low expression in both LXR drug groups (Figure 3F,G). LXR modulation could be a potential treatment strategy for ccRCC by inhibiting biomembrane synthesis.

**FIGURE 3:**
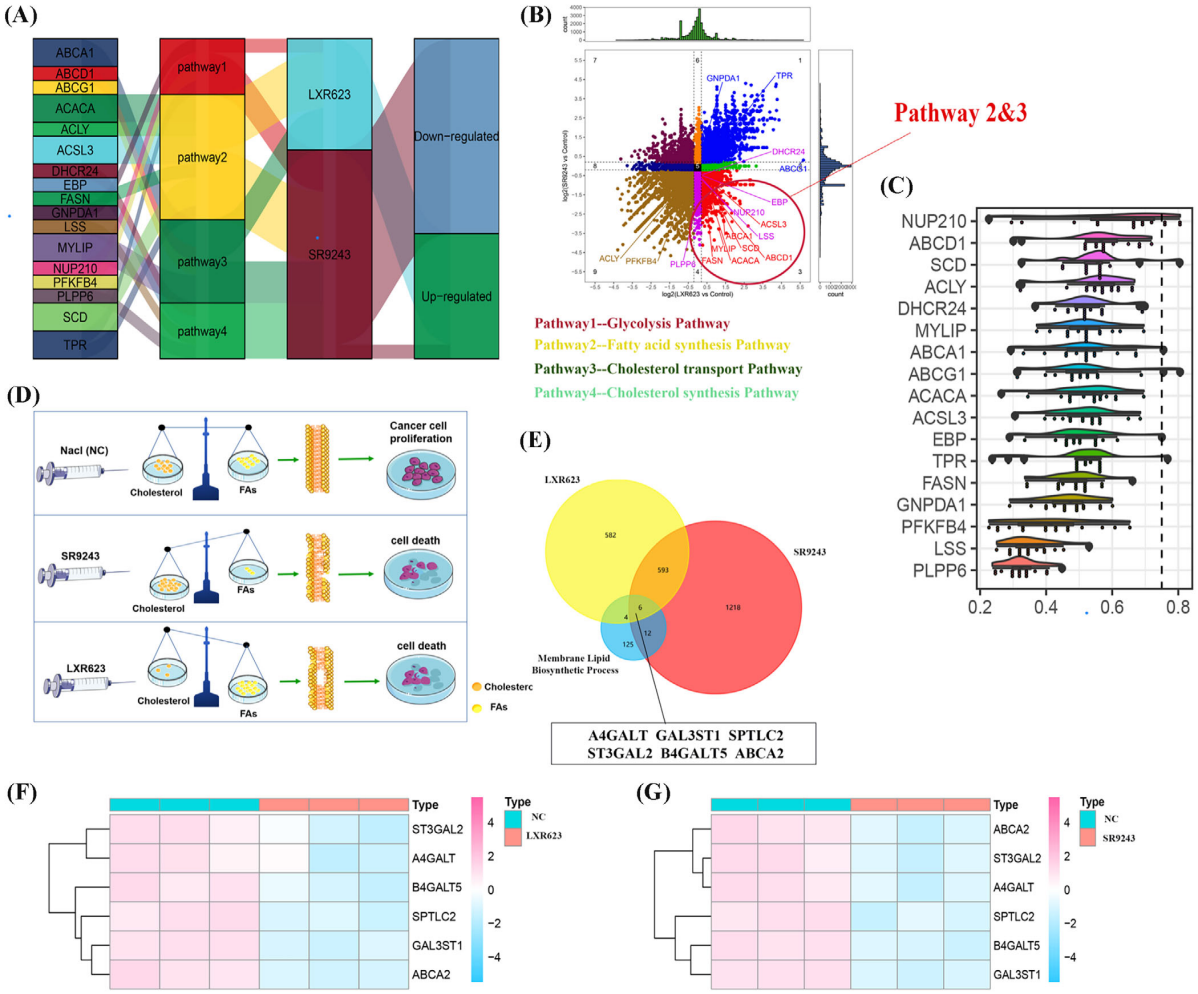
(A) The Sankey diagram shows the interrelationship between multiple factors (hub genes, four biological pathways, and drugs specifically targeting liver X receptor [LXR]). (B) The four‐quadrant graph shows the relationship between the differentially expressed genes (x‐axis) of the LXR623 treatment group and the differentially expressed genes (y‐axis) of the SR9243 treatment group, more intuitively. (C) The raincloud plot shows the different importance of these hub genes in the occurrence and development of clear cell renal cell carcinoma (ccRCC). (D) The schematic diagram shows the biological mechanism of the negative control group, the SR9243 treatment group and the LXR623 treatment group. (E) Venn diagram of the overlapping relationship between the two sets of differentially expressed genes and genes related to the membrane lipid biosynthesis process. (F) Heat map shows the expression of these six key genes between the LXR623 treatment group and the negative control group. (G) Heat map shows the expression of these six key genes between the SR9243 treatment group and the negative control group.

In conclusion, our study utilized the glycoFAsynthesis‐cholesterol synthesis axis to identify novel ccRCC subtypes, providing valuable data for future research. These results have important implications for personalized treatment strategies based on the metabolic dependencies of individual ccRCC patients. At the same time, the limitations of some research methods also have a certain impact on this experiment. For example, the comparison of metabolites such as fatty acids and sugars in ccRCC cells and ordinary cells is based on the experimental results of Hakimi et al. In the subsequent cell experiment verification part, whether the selected cell line ACHN can represent ccRCC remains controversial. Our findings suggest the potential of personalized medicine to improve outcomes and call for further investigation into metabolic dysregulation mechanisms in ccRCC.

## FUNDING INFORMATION

This project is supported by the Scientific Research Fund of the Liaoning Provincial Education Department (No. LZ2020071), the Doctoral Start‐up Foundation of Liaoning Province (No. 2021‐BS‐209), the Dalian Youth Science and Technology Star (No. 2021RQ010).

## CONFLICT OF INTEREST STATEMENT

The authors declare no conflict of interest.

## Supporting information



Supporting Information
