# Supplementary material for: The mechanism of liver X receptor regulates the balance of glycoFAsynthesis and cholesterol synthesis in clear cell renal cell carcinoma
Source: Clin Transl Med. 2023 May 3;13(5):e1248. doi: 10.1002/ctm2.1248 (PMC10157264; doi:10.1002/ctm2.1248)
Supplement: Supplementary file 1 — Supporting Information [file CTM2-13-e1248-s001.zip › Supporting Information V6.docx]

**Supplementary Materials and Methods**

**Data sources**

The metabolite data used in this study to investigate differences in key metabolites in glycoFAsynthesis and cholesterol biosynthesis pathways in clear cell renal cell carcinoma (ccRCC) tissues compared with normal kidney tissues were obtained from a study by Hakimi et al. published in Cancer Cell. This study included 138 ccRCC tissues and matched 138 normal kidney tissue samples. Our research also utilized the "omics" dataset generated by The Cancer Genome Atlas (TCGA), a collaborative project between the National Cancer Institute and the National Human Genome Research Institute that has produced a comprehensive multi-dimensional map of key genomic changes in 33 cancers. We obtained RNA-seq and clinicopathological data of these 33 types of cancer from the TCGA database. For example, the ccRCC dataset in the TCGA database comprises 539 ccRCC samples and 72 normal samples (http://cancergenome.nih.gov/). Pathway-related genes were obtained through the Gene Set Enrichment Analysis (GSEA) website (http://www.gsea-msigdb.org/gsea/index.jsp). After screening and comparison, CT images (in DICOM format) of 106 TCGA samples were obtained from The Cancer Imaging Archive (TCIA) database, which provides a publicly available repository of medical images for cancer research (https://www.cancerimagingarchive.net/).

**Cell culture and reagents**

In this study, the ACHN human renal cancer cell line was procured from the Shanghai Cell Bank of the Chinese Academy of Sciences and cultured under standard conditions in a cell incubator at 37°C, supplemented with penicillin and streptomycin. To target the LXR transcription factor, we employed two drugs, namely SR9243 (MCE, HY-16972) and LXR623 (MCE, HY-10629), both of which were obtained from a commercial source.

**Transcriptome sequencing**

Transcriptome sequencing is a high-throughput technique that enables the comprehensive identification of all transcripts present in a specific tissue or organ of a species, in a given state. This technique also allows for the analysis of various genetic features such as differential gene expression, alternative splicing, fusion genes, single nucleotide variations (SNVs), among others. In our study, total RNA was extracted from cells using the TRIzol reagent. Subsequently, mRNA was isolated and converted to cDNA using a reverse transcription kit (Takara). The RNA library was then prepared, followed by transfer to the flowcell. Finally, next-generation sequencing was carried out and data analysis was performed in accordance with Illumina standard procedures. Specifically, we conducted transcriptome sequencing of the LXR623 treatment group, SR9243 treatment group, and negative control group.

**Xenografts**

In this study, we subcutaneously injected a suspension of ACHN cells, comprising of 5×10^6^ cells, into the left armpit of nude mice. Once the tumor volume in each mouse reached a size greater than 100mm^3^, LXR623 or saline was injected. After 42 days of treatment, the mice were euthanized, and the tumors were excised for further evaluation.

**Data processing, bioinformatics analysis, and radiomics feature extraction**

In our study, we utilized the Perl and R programming languages extensively for data processing, statistical analyses, and visualization. We obtained the latest version of the official R software from CRAN (https://www.r-project.org/) and used RStudio (https://www.rstudio.com/) as our operational platform. First, we integrated gene expression levels related to the glycoFAsynthesis and cholesterol synthesis pathways to cluster clear cell renal cell carcinoma (ccRCC) patients. The resulting heat map revealed four subgroups: quiescent, glycoFAsynthesis, cholesterol, and mixed. Subsequently, we utilized the "survival" expansion package to generate survival curves for the four subgroups. In order to assess potential differences in drug sensitivity among these subgroups, we employed the pRRophetic algorithm, which utilizes GDSC and TCGA gene expression profiles to construct a model predicting drug IC50. Furthermore, we employed the TIDE and subclass mapping algorithms to predict either a single sample's response possibility or a subtype to immunotherapy. Specifically, TIDE was used to predict single-sample immune checkpoint inhibitor responses, while submap was employed to predict immunotherapy response of subtypes. Bonferroni correction was employed to adjust for the test level p-value, and the "pheatmap" expansion package was used to construct the corresponding heat map. Subsequently, we performed ssGSEA analysis to quantify the correlation between the expression levels of studied genes in the TCGA database and immune cells, utilizing the "ggplot2" and "dplyr" in the R language to generate the heat map.

Notably, differential expression of classic oncogenes and histone modification-related genes may lead to activation or inhibition of the glycoFAsynthesis and cholesterol synthesis pathways. Thus, to explore the potential regulatory mechanism of these pathways in KIRC, we generated a heat map of the expression levels of various known oncogenes involved in these pathways. Using the same approach, we also demonstrated the relationship between the glycoFAsynthesis and cholesterol synthesis pathways and two gene types involved in histone modification, SIRT and HDAC. We considered P<0.05 to be statistically significant. Finally, to identify drugs useful for targeting the glycoFAsynthesis-cholesterol synthesis axis, we employed Broad Institute's Connectivity Map Build02 (CMap), which predicts compounds that can activate or inhibit tumors based on gene expression characteristics. Using CMap, we conducted a specific analysis to investigate the mechanism of action (MoA) and drug targets related to the glycoFAsynthesis-cholesterol synthesis axis. Specifically, we employed a pattern-matching strategy based on the Kolmogorov-Smirnov test to identify similarities between differentially expressed genes (DEGs) and generate an enrichment score (ES) ranging from -1 to 1, which was then ranked according to all case data in the database. For each cancer type, we generated two tables applying the connection diagram findings to the expression characteristics of the glycoFAsynthesis-cholesterol synthesis axis, using p<0.05 as our inclusion criterion to determine the average meaningful compound for each tumor type. These compounds may inhibit or activate the glycoFAsynthesis-cholesterol synthesis axis of tumors. To process and analyze the data, we utilized the "GEOquery" package in R language to retrieve data from the Gene Expression Omnibus (GEO) database, and the "xlsx," "tidyverse," "plyr," and "circlize" packages were employed. We also generated a heatmap using "heatmap" package. Finally, we utilized the "pyradiomics" package to extract 106 features of CT images of 108 TCGA samples, including First Order Features, Shape Features, Gray Level Co-occurrence Matrix (GLCM) Features, Gray Level Size Zone Matrix (GLSZM) Features, Gray Level Run Length Matrix (GLRLM) Features, Neighbouring Gray Tone Difference Matrix (NGTDM) Features, and Gray Level Dependence Matrix (GLDM) Features.

**Statistics**

In this study, statistical analysis and graph drawing were performed using R3.6.1 software (https://www.r-project.org/) and Python3.9 (https://www.python.org/). The Kruskal-Wallis test was employed to investigate the association among multiple sets of variables, with statistical significance set at P<0.05.

**Supplementary Results**

**Immune cell infiltration analysis**

In this study, we conducted a correlation analysis of immune cell infiltration and found that the genes NUP93 and ACSL6 had a significantly positive correlation with most immune cells. Conversely, MECR and ACACB showed a strongly negative correlation with most immune cells (Fig S6A). Additionally, we investigated the expression of key oncogenes/tumor suppressor genes, SIRT family genes, and HDAC-related genes in the two patient subgroups, and generated corresponding heat maps (Fig S5 and S6B). Our results demonstrated that, compared to the cholesterol subgroup, the glycoFAsynthesis subgroup exhibited abnormally high expressions of EGFR, CTNNB1, BRAF, PTEN, KRAS, STAT3, MTOR, PIK3CA, SIRT1, and HDAC9 genes. Conversely, the cholesterol subgroup displayed abnormally high expressions of HRAS, SIRT2, SIRT3, SIRT6, SIRT7, HDAC5, HDAC6, HDAC7, HDAC8, HDAC10, and HDAC11 genes, relative to the glycoFAsynthesis subgroup.

**Variation of glycoFAsynthesis and cholesterol synthesis pathways-related genes in four ccRCC subgroups**

To investigate the copy number variation (CNV) status of pathway-related genes, statistical analysis was conducted using a heat map reflecting the mutation frequency of the four subgroups (Fig S7A), and a triangle diagram based on the mutations of the pathway-related genes in the three subgroups, namely quiescent, glycoFAsynthesis, and cholesterol (Fig S7B). Notably, ERBB4 exhibited a high CNV mutation frequency in the cholesterol subgroup, while MUC16 and ATM showed high CNV mutation frequency in the glycoFAsynthesis subgroup. Additionally, combining the mutational information of ERBB4 and VHL revealed that the expression levels of cholesterol-related genes were lower in the gene double-loss group compared with other groups (Fig S7C). Similar results were obtained when combining the mutation information of ERBB4 and PBRM1 (Fig S7D). Furthermore, we analyzed the top 20 genes with SNV frequencies in ccRCC and identified VHL, PBRM1, TTN, SETD2, and BAP1 as having mutation frequencies of more than 10% (Fig S2).

To explore the specific relationship between the cholesterol synthesis pathway and ccRCC patients, we constructed a cluster analysis model based on the mRNA expression of genes related to the cholesterol synthesis pathway. Based on the final cluster analysis results, the patient samples were categorized into four groups, namely cluster 1, cluster 2, cluster 3, and cluster 4 (Fig S7E-F). Notably, the HSD17B7 gene showed a significant up-regulation trend in all cluster groups. In cluster 3, most of the genes related to the cholesterol synthesis pathway exhibited a significant down-regulation trend. Subsequently, we utilized the clinical survival information of the four clusters to generate the corresponding survival curve, with the worst prognosis observed in the ccRCC patients in the cluster 3 group (Fig S7G). Furthermore, we conducted a similar cluster analysis based on glycoFAsynthesis pathway-related genes, resulting in the division of ccRCC into cluster 1, cluster 2, cluster 3, and cluster 4 (Fig S3A). The survival curve showed that cluster 3 had the worst survival rate (Fig S3C). Moreover, cluster 3 exhibited more abnormal activation of genes than other clusters. The box plot revealed that the enrichment scores of the four clusters ranged from high to low, namely cluster 4, cluster 3, cluster 1, and cluster 2 (Fig S3B).

**Analysis of hub genes and CMap analysis in multiple types of tumors**

In this study, we investigated 17 hub genes in various cancer types using data from the TCGA pan-cancer project. We analyzed CNV, SNV, co-expression, and gene expression levels and overall survival rates (Fig S8A-E) and found that KICH, OV, and UCS exhibited a higher frequency of CNV gain/loss. The TPR gene had a higher frequency of CNV gain in many tumors (Table S5), while the PFKFB4 gene had a higher frequency of CNV loss in several tumors (Table S6). Although most genes showed low SNV frequencies across different tumors, KICH, OV, and UCS displayed higher SNV frequencies (Table S7). Co-expression analysis revealed a strong positive correlation between ACACA and PLPP6, as well as between ACACA and FASN. Heat maps of Log2FC gene expression levels between normal and cancer tissues revealed that most hub genes exhibited higher expression levels in most cancers (Table S8). The overall survival analysis results indicated that most genes played an oncogenic role in tumor occurrence and development (Table S9).

To identify potential compounds and inhibitors that might target hub genes, we used CMap, a data-driven approach to discover links between genes, chemicals, and biological situations (Fig S9). Additionally, we utilized the "ggstatplot" package in the R language to investigate the relationship between cholesterol biosynthesis genes score and immune cell infiltration (Fig S4A). We found a negative correlation between cholesterol biosynthesis genes score and various immune cell infiltration, such as MHC class I and cytolytic activity (Fig S4B-C). Similarly, analysis of the score of glycoFAsynthesis genes and immune cell infiltration revealed a negative correlation between the two, including Type Ⅱ IFN response and Treg (Fig S4D-F).

**Construction and evaluation of a radiomics prediction model for G-M-C subtype clustering**

In this study, we utilized machine learning algorithms to construct a prediction model for G-M-C subtype clustering based on 108 radiomics features extracted from CT images. To begin, we calculated the Spearman rank correlation between the 108 features and displayed the results using a heat map (Fig S10A-B). Features with a correlation greater than 0.9 were selected for inclusion in the model construction. Next, the LASSO regression algorithm was employed to further screen the coefficients, resulting in the selection of nine radiomics features for the model (Fig S11A-B). The proportion of the types of all features is depicted in a fan diagram (Fig S11C).

The final prediction model was established using the following formula: Label = 1.190829794508306 + 0.052956 * original_firstorder Maximum + (- 0.159104) * original firstorder Skewness + (- 0.027055) * original glcm Inverse-Variance + (- 0.103792) * original glcm MCC + 0.128839 * original gldm Small-Dependence-Low-Gray-Level-Emphasis + (-0.003256) * original glrlm Long-Run-Low-Gray-Level-Emphasis + 0.043300 * original glszm Size-Zone-Non-Uniformity + 0.113057 * original glszm Zone-Percentage + (- 0.049735) * original shape Flatness (Fig S11D). Here, label 0 represents the G cluster, label 1 represents the M cluster, and label 2 represents the C cluster. The performance of the model was evaluated using six different machine learning algorithms, including support vector machine (SVM), K-Nearest Neighbor (KNN), random forest, Extratrees, XGboost, and LightGBM, with the results demonstrating that the model based on Extratrees had the best performance. Evaluation indicators, including accuracy (Fig S11E-F), AUC (Fig S11G-I), and the confusion matrix (Fig S11J-L), were displayed.

**GlycoFAsynthesis and cholesterol synthesis in ccRCC**

Currently, mounting evidence suggests that ccRCC can be classified as a metabolic disorder. This is due to the observation that ccRCC specimens contain high levels of glycogen, cholesterol, and lipids. Lipid droplets are dynamic organelles that are crucial in the regulation of lipid absorption, distribution, and utilization. The accumulation of lipid droplets is a common feature observed in many cancers, particularly in cells exposed to hypoxia or nutrient deficiencies. The most exemplary representation of such accumulation is evident in ccRCC, where numerous lipid droplets give the cells a transparent appearance. The ratio of lipid content in ccRCC is significantly higher than other tumors, implying that lipid metabolism plays a vital role in the development and progression of ccRCC. Fatty acids and cholesterol, the main components of lipid metabolism, form a flexible feedback loop that regulates and interacts to meet the metabolic demands of cancer cells.

The de novo synthesis pathway for fatty acids is crucial for providing the lipids necessary for the growth of tumor cells. Previous research has revealed that lipogenic enzymes and endogenous fatty acid synthase (FASN) are highly expressed in cancerous tissues, whereas normal cells primarily obtain fatty acids from exogenous sources. Fatty acids are synthesized from the Warburg metabolite acetyl-CoA through FASN, stearoyl-CoA desaturase (SCD1), and other downstream glycolytic enzymes. Upregulation of FASN and SCD1 has been associated with various forms of cancer. Recent studies have shown that inhibitors of adipogenesis can impede the activities of FASN, SCD1, and SREBP-1c, leading to reduced proliferation of cancer cells and apoptosis. Thus, fatty acid anabolism plays a vital role in the development of ccRCC. However, cholesterol levels in ccRCC tissues, a necessary raw material for rapid tumor growth, are significantly higher than in normal kidney tissues. The contradictory phenomenon is that our previous studies have found that the expression of key enzymes for synthesizing cholesterol, such as HMGCR and DHCR24, is lower in ccRCC tissues than in normal tissues. Hence, a complex metabolic regulation network must exist in ccRCC. To investigate the underlying mechanism for a unique phenomenon, we categorized the metabolic processes of glucose and lipid metabolism into glycolysis-fatty acid synthesis and cholesterol synthesis pathways. Through bioinformatics clustering analysis, we aimed to identify potential factors involved. Previous research revealed that the levels of glycolysis and fatty acid synthesis metabolites, including glucose-6-phosphate, fructose-6-phosphate, lactate, stearate, oleate, palmitate, and palmitoleate, as well as cholesterol content, were higher in ccRCC tissues than in normal tissues. Upon further analysis, we observed that patients in the glycoFAsynthesis group had a significantly poorer prognosis compared to other groups, while patients in the cholesterol synthesis group had a better prognosis. In particular, patients in the T1+T2 and G1+G2 groups who belonged to the cholesterol synthesis group had better prognoses. At the same time, patients in the T3+T4 and G3+G4 groups who belonged to the quiescent group had better prognoses, while those belonging to the cholesterol synthesis and glycoFAsynthesis groups had worse prognoses. These results suggest that cholesterol synthesis may play a protective role in early-stage and low-grade patients, while it may act as a risk factor in advanced-stage and high-grade patients. This complex phenomenon makes us more interested in the particularity of metabolism in ccRCC. Are there some regulatory factors involved in glycolysis and the metabolism of fatty acids and cholesterol, which finally led to the occurrence of the above situation? Does fatty acid and cholesterol have lipotoxicity? Does cholesterol or fatty acid content in tumor cells, which are too high or too low cause tumor cells to lose their malignant phenotype and the ability to grow rapidly? Is there a balance of the above metabolites in tumor cells, and if the balance is disrupted, will it inhibit tumor progression? To this end, we will examine the nuclear transcription factor receptor "LXR" and its potential role in regulating metabolism in ccRCC.

**Experimental details and related parameters**

Medication name: LXR623.

Route of administration: Intraperitoneal injection.

Dosage of medication: 58mg/kg, with each 20g of body weight requiring 1.16mg of the drug.

Nude mouse information: 4-week-old SPF-level mice.

Duration of drug treatment: 6 weeks.
