# Supplementary figures and images for: The mechanism of liver X receptor regulates the balance of glycoFAsynthesis and cholesterol synthesis in clear cell renal cell carcinoma
Source: Clin Transl Med. 2023 May 3;13(5):e1248. doi: 10.1002/ctm2.1248 (PMC10157264; doi:10.1002/ctm2.1248)

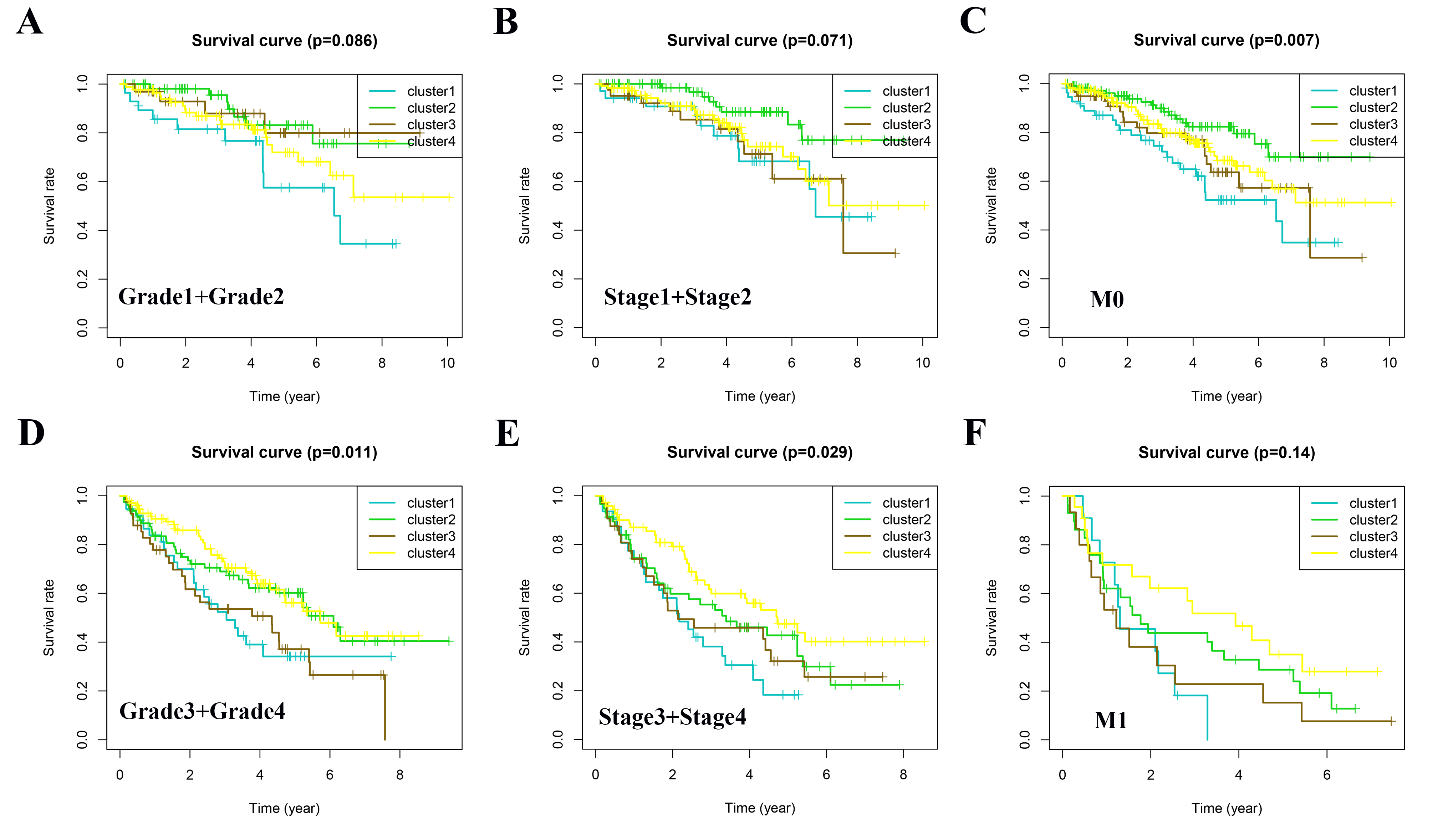

Supplement: Supplementary file 1 — Supporting Information [file CTM2-13-e1248-s001.zip › Figure S1.tif]

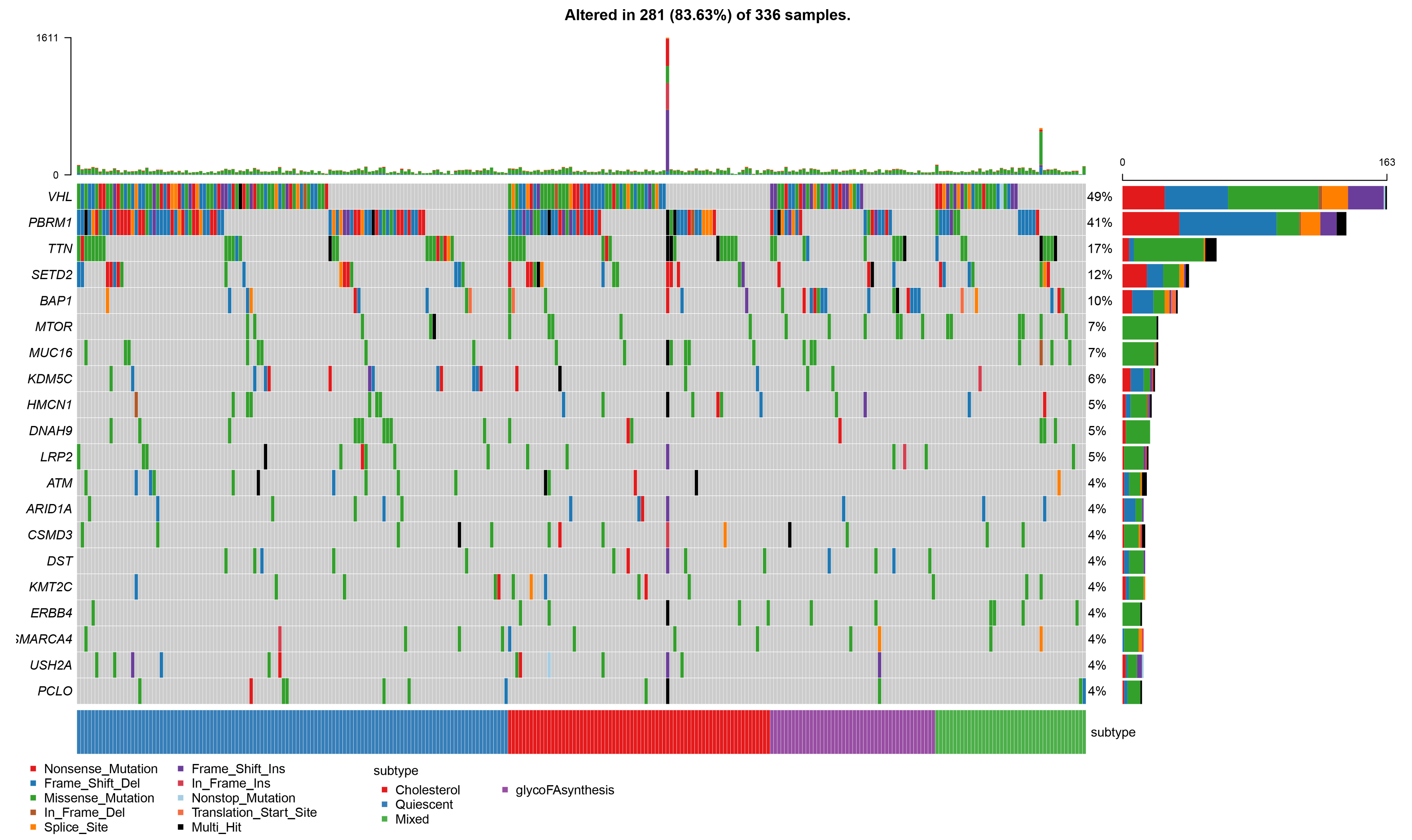

Supplement: Supplementary file 1 — Supporting Information [file CTM2-13-e1248-s001.zip › Figure S2.tif]

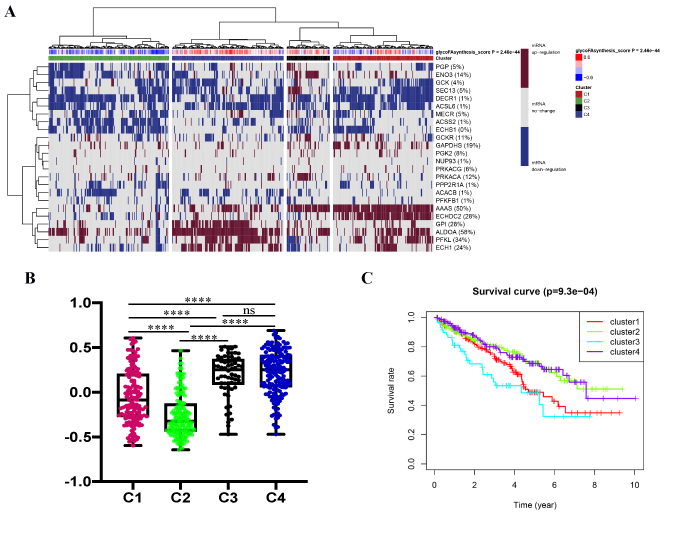

Supplement: Supplementary file 1 — Supporting Information [file CTM2-13-e1248-s001.zip › Figure S3.tif]

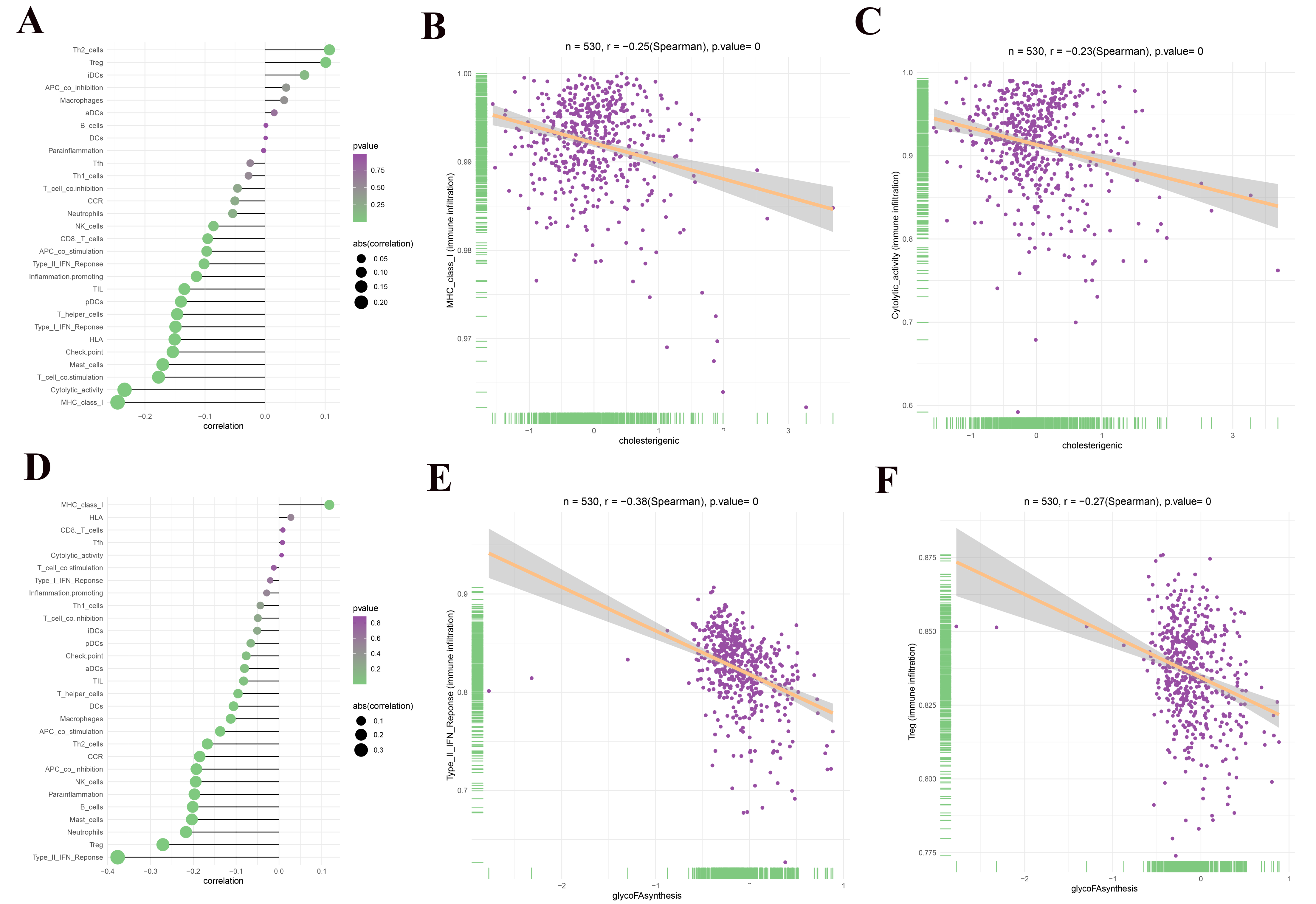

Supplement: Supplementary file 1 — Supporting Information [file CTM2-13-e1248-s001.zip › Figure S4.tif]

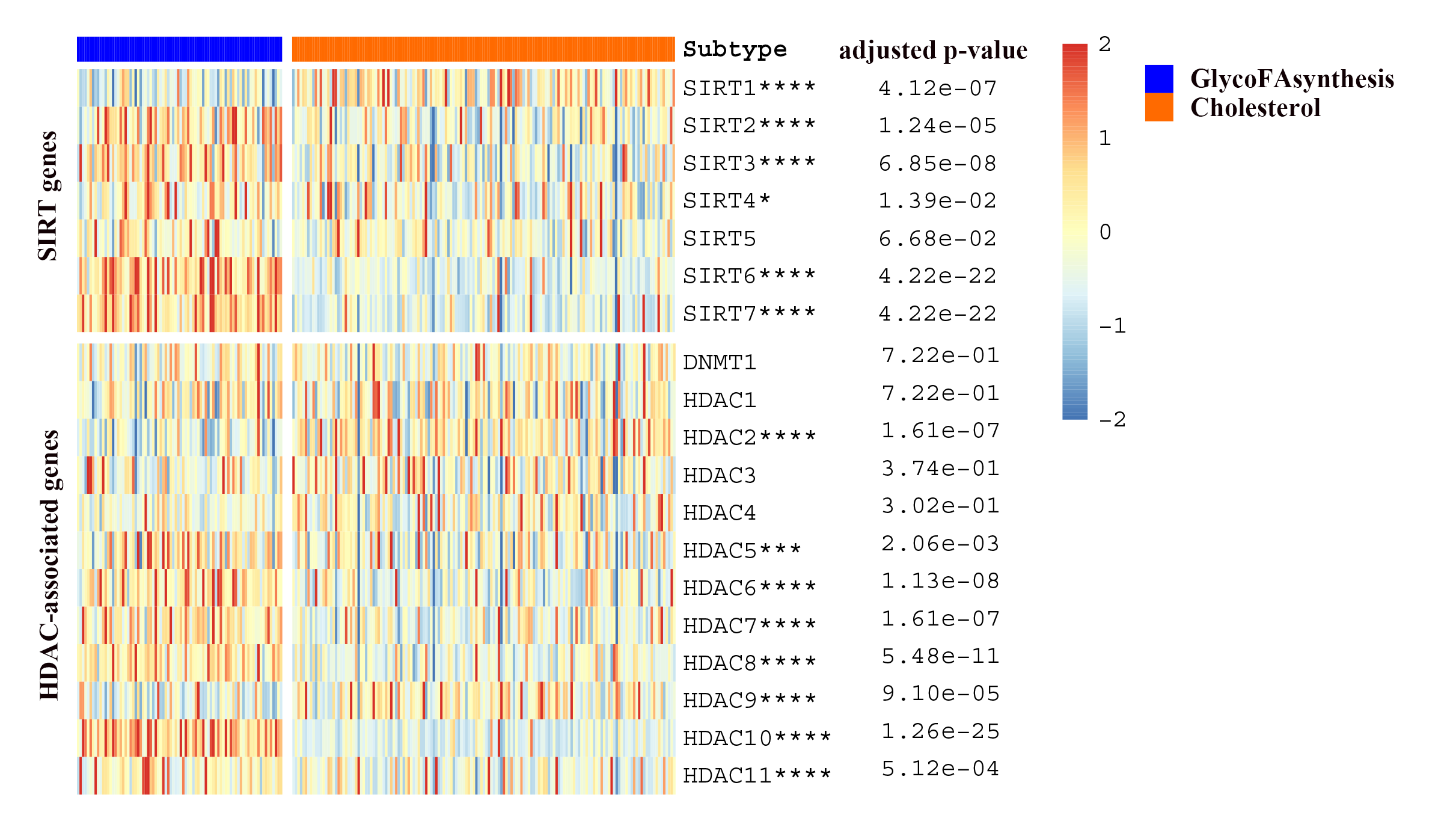

Supplement: Supplementary file 1 — Supporting Information [file CTM2-13-e1248-s001.zip › Figure S5.tif]

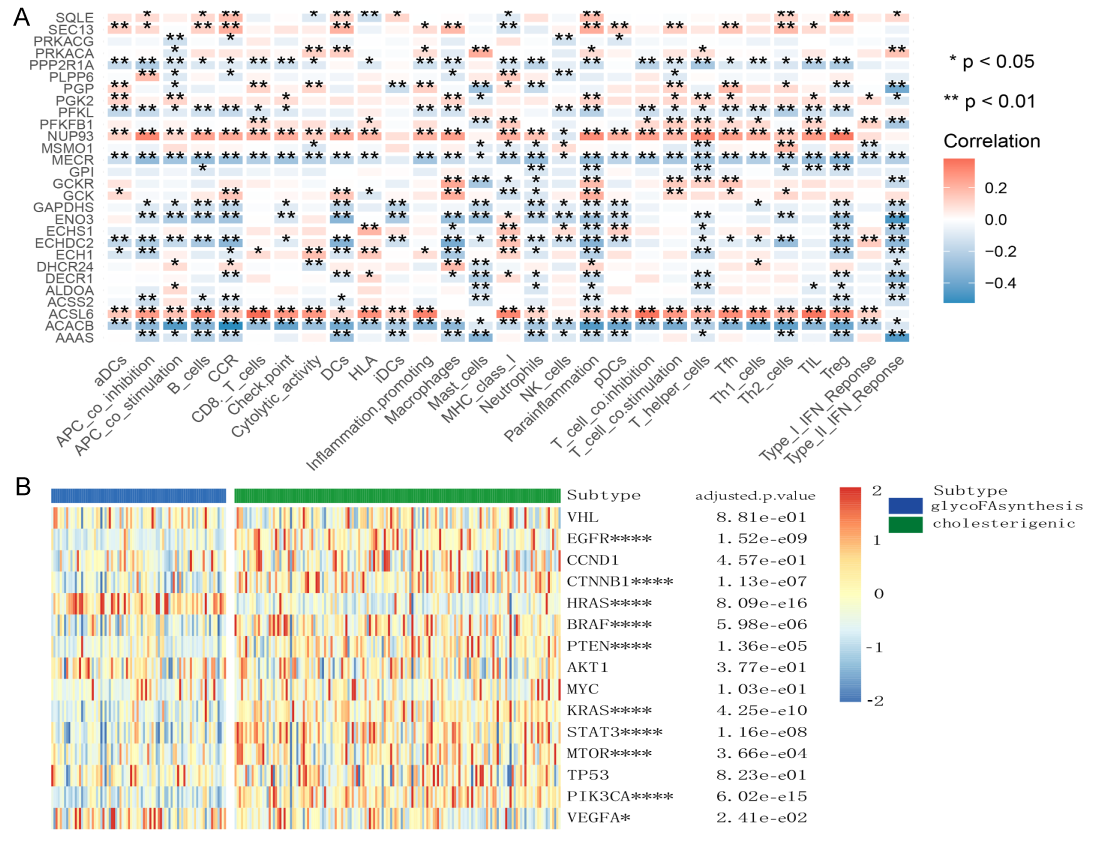

Supplement: Supplementary file 1 — Supporting Information [file CTM2-13-e1248-s001.zip › Figure S6.tif]

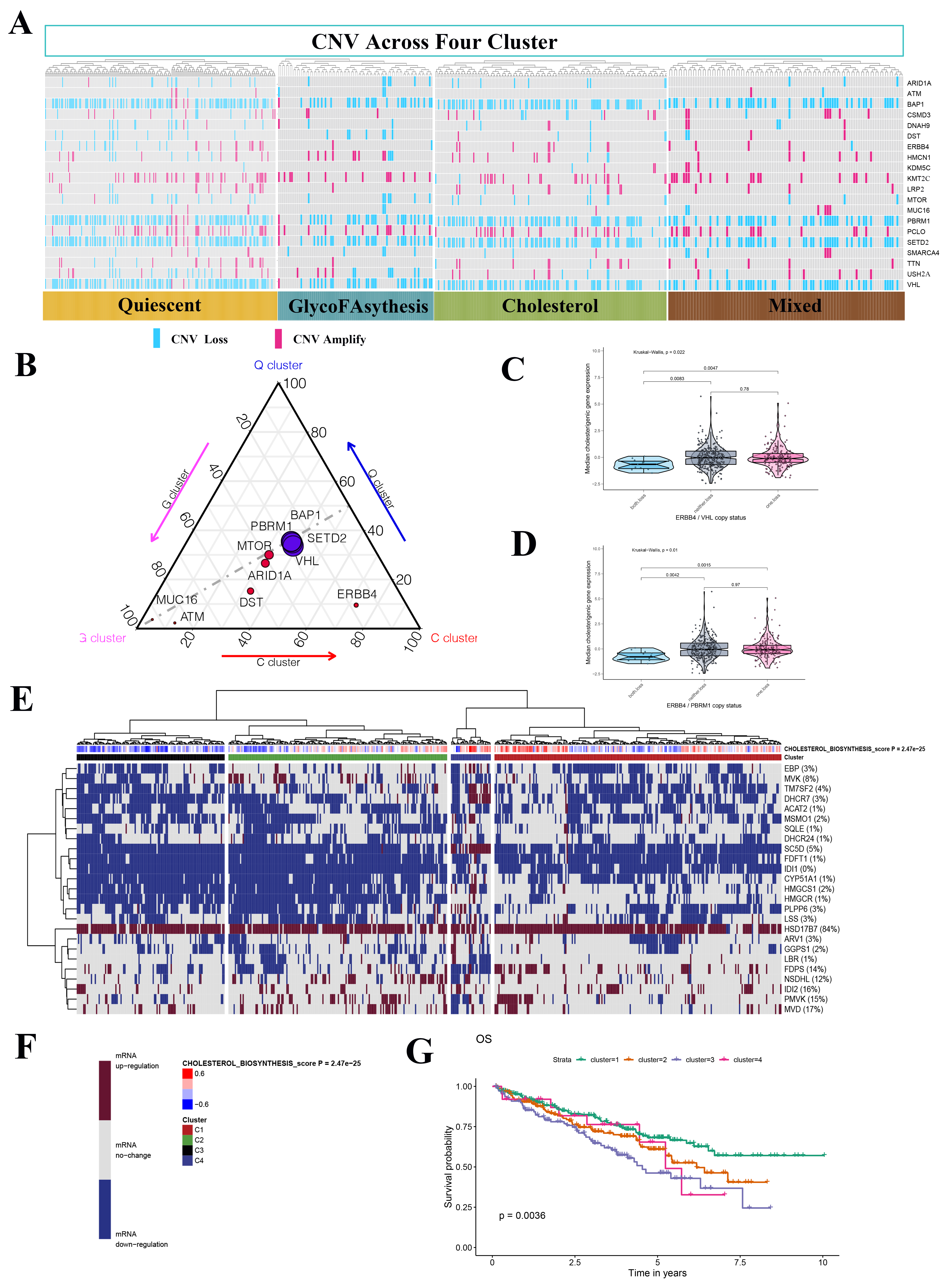

Supplement: Supplementary file 1 — Supporting Information [file CTM2-13-e1248-s001.zip › Figure S7.tif]

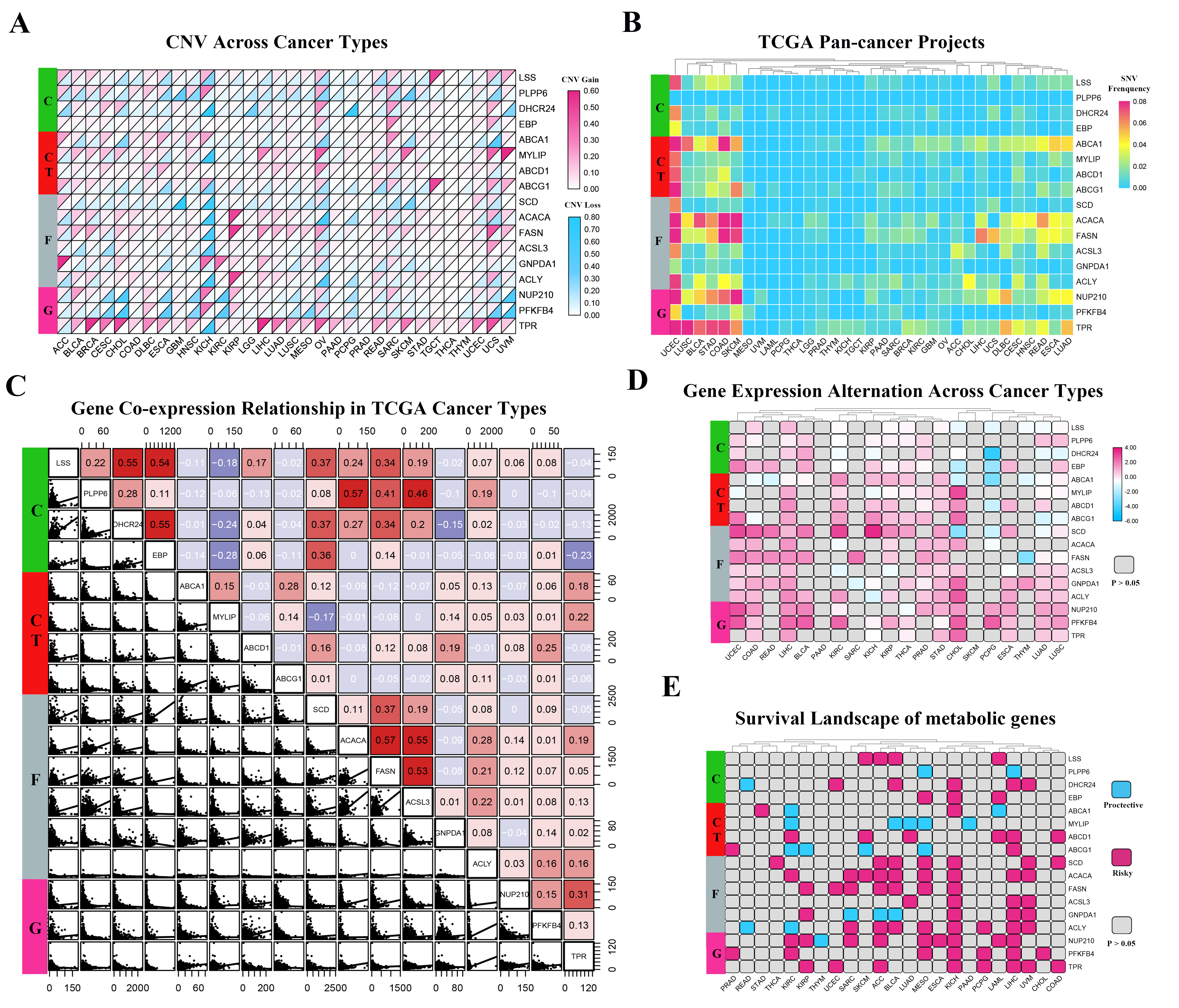

Supplement: Supplementary file 1 — Supporting Information [file CTM2-13-e1248-s001.zip › Figure S8.tif]

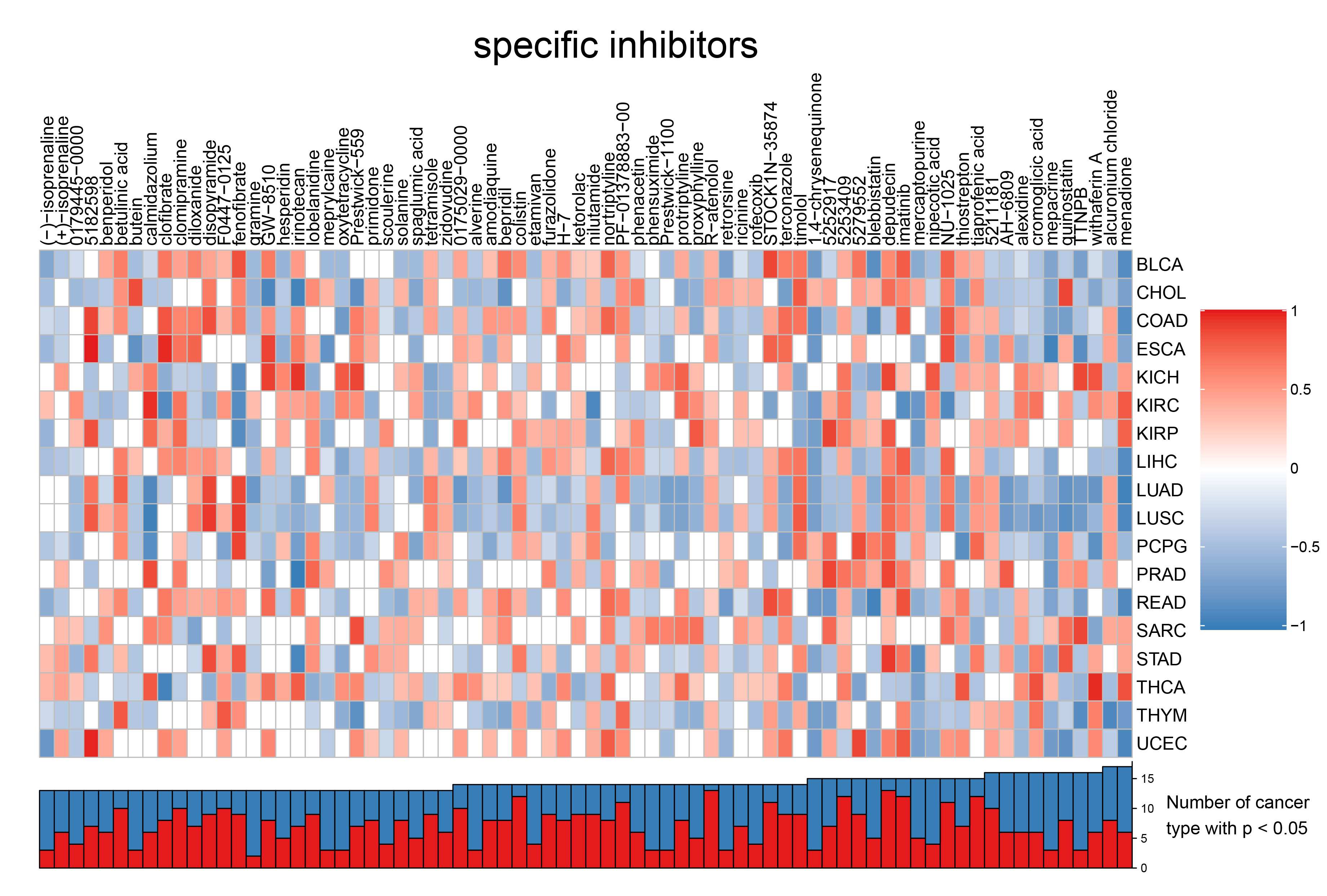

Supplement: Supplementary file 1 — Supporting Information [file CTM2-13-e1248-s001.zip › Figure S9.tif]
